# Decontamination of Intravaginal Probes Infected by Human Papillomavirus (HPV) Using UV-C Decontamination System

**DOI:** 10.3390/jcm8111776

**Published:** 2019-10-24

**Authors:** Maxime Pichon, Karine Lebail-Carval, Geneviève Billaud, Bruno Lina, Pascal Gaucherand, Yahia Mekki

**Affiliations:** 1 Bacteriology and Infection Control Laboratory, Infectious Agents Department, University Hospital of Poitiers, 86021 Poitiers Cedex, France; 2 Hospices Civils de Lyon, Département de chirurgie gynécologique, Hôpital Femme-Mère-Enfant, 69000 Lyon-Bron, France; karine.le-bail-carval@chu-lyon.fr (K.L.-C.); pascal.gaucherand@chu-lyon.fr (P.G.); 3 Hospices Civils de Lyon, Laboratoire de Virologie, Institut des Agents Infectieux, Groupement Hospitalier Nord, 69317 Lyon Cedex 04, France; genevieve.billaud@chu-lyon.fr (G.B.); Bruno.lina@chu-lyon.fr (B.L.); Yahia.mekki@chu-lyon.fr (Y.M.)

**Keywords:** HPV, genital cancer, intravaginal ultrasound, disinfection, ultraviolet disinfection

## Abstract

Background: This three-step study evaluated ultraviolet-C (UV-C) efficacy against human papillomavirus (HPV) found on vaginal ultrasound probes. Methods: The first two steps evaluated UV-C disinfection of vaginal ultrasound probes in routine condition. During the first phase, the probe (*n* = 100) was sampled after a complete cleaning and disinfection protocol, i.e., cleaning with chemically impregnated wipes, followed by UV-C. During the second phase, the probe (*n* = 47) was sampled after cleaning and UV-C. The final step consisted of applying mixes of HPV on a dedicated, covered probe (*n* = 15) then sampling the cover, the probe after removal of the cover, after cleaning, and after UV-C. HPV detection was performed using CLART^®^ HPV2 PCR (Genomica, Madrid, Spain). Results: In the first phase, no probes were found to be positive for both DNA after UV-C. In the second phase, eight probes were found to be positive after cleaning (seven with human DNA and one with HPV) and negative after UV-C. In the final phase, one probe was found to be positive for HPV for each sample except after UV-C. Conclusions: Covers followed by a chemically impregnated wipe are not sufficient to ensure patient safety during vaginal ultrasound examinations. UV-C is effective in routine conditions against contaminations found on vaginal ultrasound probes, especially HPV.

## 1. Introduction

Ultrasound is a physically safe imaging modality, widely accepted and used across almost all medical specialties [1].

Infection control in ultrasound is rather new, however, even for endocavity probes. Several studies have looked at probe cover perforations, but few observed actual contamination of probes [2,3,4]. In 2010, Kac et al. were pioneers in evaluating the presence of human papillomavirus (HPV) on endocavity probes despite usage of disposable covers. Their reports opened the way for further studies showing contamination of ultrasound probes with HPV despite usage of disposable probe covers followed by low-level disinfection (LLD) [5,6,7]. A group of public health leaders stated in 2015 that “until recently, endocavity ultrasound has not been regarded a high-risk procedure regarding infection transmission”. However, following various current reports of contaminated transvaginal ultrasound probes, this has changed [8].

The identification and quantification of patient infections linked to widespread, below-standard disinfection of endocavity ultrasound probes is severely limited since ultrasound is largely an outpatient practice. Also, the most potential infections are, by nature, sexually transmitted infections (STIs). Leroy et al. performed mathematical modeling and found that 72,000 potential healthcare-associated infections (HAIs) are due to vaginal and rectal ultrasound exams for a baseline of 4 million exams, which is about the yearly number for France [9]. This work was challenged by Bénet et al., who performed a retrospective analysis of 8232 and 13,730 patient files looking for the seroconversions of hepatitis C (HCV) and human immunodeficiency virus (HIV), respectively, that could originate from endocavity ultrasound [10]. They found no seroconversions for HIV and one for HCV. In 2008, the French National Institute for Health (“Institut de Veille Sanitaire”) tested 472 patients who had undergone endocavity ultrasound with poor hygiene conditions and found three infected patients, each with a different infection (Hepatitis B or C virus, or syphilis) without the possibility to conclude if the infection was due to the ultrasound exams [11]. National Health Society (NHS) of Scotland recently published an epidemiological study indicating that endocavity ultrasound without high-level disinfection (HLD) was associated with an increased risk of infection [12].

Nevertheless, guidelines and studies pushing the implementation of systematic HLD have recently flourished in disseminating the level of disinfection that was traditionally recommended in the United States, Canada, Australia, Scotland, Wales, Israel, Germany, England, and Ireland and by the World Federations of Ultrasound and the European Society of Radiology. Therefore, it appears that HLD is now the international standard for endocavity ultrasound probes.

From a practical point of view, the HLD of transducers is currently poorly followed, at least in Europe [13]. Education as well as technical constraints (time and toxicity) for available HLD solutions prevent healthcare professionals from complying with current worldwide practices to ensure patient safety in their ultrasound practice [14]. Consistently performing HLD of ultrasound probes with chemical soaking is recognized as being so difficult that properly doing so is congratulated with awards. The usual techniques for HLD present such practical drawbacks that lack of compliance could be seen as a reasonable trade off by many ultrasound users unaware of the infection transmission risk [15]. In this context, rapid HLD based on UV-C irradiation (with a biocidal peak at 254 nm) is considered desirable by healthcare professionals [16].

HPV recently appeared as the main issue for vaginal ultrasound probe disinfection because of its prevalence and consequences. It has also been discovered that few HLD products are actually efficient against HPV [16,17]. UV-C has been shown to be efficient against HPV in laboratory and routine conditions with a five-minute-long cycle when a full protocol is applied [6,18]. However, UV-C efficacy needed to be further investigated since the current version of the UV-C system has a much shorter cycle duration (90 seconds-long) that was not previously studied against real HPV contamination. Furthermore, the role of UV-C alone in complete cleaning and disinfection protocol was not comprehensively studied as well.

The aim of our study was to determine if UV-C disinfection is efficient in routine conditions, especially against HPV.

## 2. Methods

### 2.1. Study Design and Materials

The study was conducted during clinical consultations of the Gynecology department (University Hospital of Lyon, France) on randomly included patients in December 2013. It has been carried out in three successive phases described thereafter. 

The study focused on the evaluation of Antigermix^®^ S1 (AS1), a UV-C disinfection chamber designed by Germitec (Germitec, Ivry-sur-Seine, France). AS1 uses six low-pressure tubes and two photodiodes to control the UV-C disinfection cycle that terminates when the sensors have measured a targeted dose. The dose programmed for AS1 cycle was 293 mJ/cm^2^ for 90 s.

Vaginal ultrasound probe cables were equipped with an RadioFrequency Identified (RFID) collar to work with AS1 to ensure their identification, which is needed for the UV automaton to launch a cycle and secure the probe positioning within AS1 chamber. The probes were used with a condom as a disposable cover (Sagami Rubber Industries, Atsugi, Japan) and cleaned before disinfection using only one chemical (Quaternary Ammonium-based formulation) impregnated wipe (Sani-Cloth Active^®^, PDI Healthcare, Orangeburg, SC, USA). The study primarily focused on HPV detection on vaginal ultrasound probes that were performed by PCR using the CLART^®^ HPV2 kit (Genomica, Madrid, Spain). 

This study also aimed to compare two protocols usually found in infection prevention practice of endocavity ultrasound probes. The simple protocol consisted of performing LLD with a chemically impregnated wipe on a probe used with a disposable cover. The more rigorous protocol consisted of performing HLD on a visibly clean probe. 

#### 2.1.1. First Phase

This first phase was a feasibility phase to evaluate AS1 efficacy in routine conditions with vaginal ultrasound probes. The study observed DNA presence (HPV and human DNA) after the complete hygiene protocol was performed, i.e., the disposable probe cover was removed, the probe was cleaned (soil removal as gel and eventually visible body fluids) with one Sani-Cloth Active^®^ wipe (Sani-Cloth Active^®^, PDI Healthcare, Orangeburg, SC, USA), and the probe was finally disinfected using AS1.

The probes were used in routine clinical conditions without patient selection. Probes disinfected by AS1 (*n* = 100) were randomly selected to be analyzed. For the analysis, the probes were sampled on their whole surface using dry swabs that were then tested by PCR using the kit CLART^®^ HPV2 (Genomica, Madrid, Spain). 

#### 2.1.2. Second Phase 

The second phase was similar to Phase l and focused on observing the relative efficacy of cleaning (i.e., usage of our wipes following disposable probe cover removal) and AS1. For this purpose, 47 probes (*n* = 47) were sampled three times at different steps of the full cleaning and disinfection protocol. Swabs were applied (i) on the external surface of the cover before its removal; (ii) on the probe after the cover was removed and the probe cleaned with Sani-Cloth Active^®^ wipes (except for the region sampled in the third sample); and (iii) on the probe after its disinfection by AS1. Note that this third specimen was sampled on a different region of the probe than the one cleaned with wipes to evaluate the benefit to use AS1 solely.

All samples were then tested using the routine laboratory method previously mentioned.

#### 2.1.3. Third Phase

Finally, to better understand the mechanism of vaginal ultrasound probe contamination and the efficacy of UV-C disinfection on HPV-contaminated probes, dedicated probes, covered with a disposable sheath, were artificially contaminated. To do so, HPV clinical samples routinely received at the laboratory were used in this phase. Cytology samples containing contaminated cells (*n* = 15) were discharged in 1 mL of Eagle’s Minimal Essential Medium. Selected samples were of different types (five mono-infected samples, four bi-infected samples, five multi-infected samples, one HPV-negative sample, Table 1), and a volume of 750 µL of this contaminated medium (or an HPV-negative sample for control) was applied to the covered ultrasound probes. The integrity of the HPV types was validated by a positive PCR on the remaining volume after contamination (and a negative result for the negative sample). Before sampling for this phase, the contaminated medium applied on the probe was completely dried at room temperature then visually inspected. This process allows mimicking of a “natural” contamination by vaginal fluids.

After contamination of each medium, four consecutive samples were performed for each probe: (i) on the external surface of the probe cover; (ii) on the probe before wipe cleaning, (iii) on the probe after wipe cleaning (Sani-Cloth Active^®^, PDI Healthcare, Orangeburg, SC, USA) (except for the region sampled in the fourth sample); and (iv) after disinfection with AS1. Samples were tested using the routine laboratory method previously mentioned.

During the third phase, any probe that was intentionally loaded with HPV during investigations were returned to the manufacturer after extensive disinfection (including DNA and viral decontamination using laboratory decontaminant solutions).

### 2.2. HPV Testing

All samples were tested by PCR using the CLART^®^ HPV2 PCR kit (Genomica, Madrid, Spain) according to manufacturer’s recommendations. This molecular test detected up to 35 different low- and high-risk HPV genotypes (17 low-risk HPV 6,11,40,42,43,44,54,61,62,70,71,72,81,83,84,85,89 and 18 high-risk HPV 16,18,26,31,33,35,39,45,51,52,53,56,58,59,66,68,73,82). Sampling and assays were validated using internal controls (human cellular detection and amplification control). An external positive control, part of an external control procedure, was added to each experiment. To assess the absence of contamination, a no-template negative control, a mixture of water and human DNA, was tested at the same time as other samples. 

### 2.3. Statistical Analysis

Comparison between remaining DNA after each of the two disinfection protocols (LLD and HLD) mentioned above was performed using Fisher’s exact test on Graph Prism v7.00 (GraphPad Software Inc., San Diego, CA, USA). *p* < 0.05 was considered significant.

### 2.4. Ethics Statement

All samples obtained during the usual clinical management of the patients were analyzed anonymously. Due to the type of study (performance evaluation of disinfection systems), no clinical data were collected (except for biological data). No additional samples were taken for this study, and studies on clinical samples used for diagnosis were approved by the local ethical committee on 7 July 2013. All samples were de-identified prior to this study.

## 3. Results

### 3.1. First Phase

No probe (*n* = 100) was found positive for HPV or human cellular DNA after AS1. External controls (mixes of viral and human DNA) were verified positives. This phase suggested the efficacy of UV-C disinfection on clinically used vaginal ultrasound probes. By degrading human and viral DNA, second and third phases were allowed to be performed. 

### 3.2. Second Phase

All the 47 probes covers were intact (not visibly damaged) and positive for human and HPV DNA. After probe cover removal and wipe cleaning, eight probes were positive for human or viral DNA (seven probes with human DNA and one with a high-grade HPV-51). After UV-C disinfection, all probes were found to be negative. The HLD procedure was found to be statistically more efficient than LLD (seven contaminated probes versus no contaminated probe, *p* < 0.05). The efficacy of the disinfection procedures is summarized in Table 2 (raw results in Table S1).

### 3.3. Third Phase

All 15 probe covers were intact and found to be positive for human DNA and HPV DNA (except for the negative sample, which was only positive for human DNA). After probe cover removal, one probe was positive for high-risk HPV 16. This HPV contamination remained after LLD and was further eliminated with AS1 disinfection (*p* < 0.05; raw results in Table S2).

## 4. Discussion

This study is, to our best knowledge, the first to evaluate UV-C disinfection efficacy against HPV in real conditions when the UV-C system is run within a short time cycle (90 s). UV-C is the only system that has been evaluated against HPV in real conditions, as other HLD products and systems have been evaluated only in laboratory conditions. The results of this study indicated that UV-C can be used to ensure patient safety during ultrasound examinations. During the first phase, if probes were not verified as contaminated before disinfection, it can be reasonably assumed that they were contaminated by HPV given the very consistent results found in the literature on this topic [6,7]. 

The second and third parts confirmed that the LLD procedure alone is unsafe and that HLD, when using a method that can reliably inactivate HPV, is strongly recommended to ensure patient safety. Artificial contamination methods using cytology samples containing contaminated cells provided important data on the effectiveness of the tested procedure. This method created similar situations to what the probe would be covered by during an ultrasound probe procedure. This method allows better characterization of these performances than the use of processed viral DNA extracts without protection from cellular keratins or viral capsid. Our evaluation of AS1 showed that UV-C disinfection was effective against HPV in routine conditions for vaginal ultrasound probes. 

This study took place in a high-turnover gynecological practice (12,000 emergency consultations with 8000 vaginal ultrasound examinations annually) in a specialized university hospital. UV-C disinfection is very helpful for busy healthcare professionals to comply with HLD regulation because of its speed (90 s), simplicity (push-button processes without consumables nor color indicators to check, as disinfection verification is automatically performed by optical sensors), and absence of toxicity (no chemical remnants or emanation). 

Further studies (larger and multicentric) must be performed before employing the UV-C system in clinical practices. It has, for example, been demonstrated that hydrogen peroxide vapor systems are at least as effective as UV-C disinfection [19]. Nevertheless, the authors of this study insisted on the benefit of using the UV-C system, as this system remained easy to use, effective, safer, and faster than other decontamination systems. Evaluations of the kill-rate of these systems are needed to demonstrate the benefit of using one system over another. Moreover, the optimized time of UV exposure must be evaluated to ensure quick and complete decontamination of the probe independently to its clinical use. Furthermore, this study highlighted the need for standardized solutions that include HPV. These solutions will allow complete evaluation of the decontamination power for UV-C solutions similarly to bacterial spore suspensions.

Finally, this study has shown that condoms used as ultrasound probe covers could present the residual rate of leakage. Consequently, these leakages lead to a potential for transmission of HPV by ultrasound examination if inadequate protections are used. Guidelines from the American Institute of Ultrasound in Medicine recommend to cover the probe by a single-use barrier (preferably a non-lubricated, non-medicated condom), even when the probe is completely cleaned after examination [3]. These guidelines highlighted the high leakage rate of commercial probe covers (8% to 81%) compared to condoms as used in our study (0.9–2%) [20,21]. The use of condoms, compared to commercial probe covers, lead to a 10- to 100-fold difference in viral load reduction (0.1–11 log_10_ reduction versus a 1.7–2.0 log_10_ reduction). These data, supported by our study (even if this study has not focused on the barrier protection during examination), have to be explained to patients, and women have to be warned about a potential transmission during endovaginal ultrasound.

## 5. Conclusions

Even if the sample size of this study is too low to allow for a major decision concerning the choice of a disinfection system associated with adequate “viral barriers” use, high-level disinfection, including efficacy against HPV, has become the international standard for endocavity ultrasound probes. UV-C has been confirmed as a relevant and adapted solution and we believe it would be quite useful that scientific societies, insurances, healthcare technology agencies, and other governmental competent bodies provide guidance to ultrasound users in understanding the financial costs (both explicit and hidden) of evaluations, as ultrasound probe HLD is not cost-neutral; that the number of ultrasound examinations is quite high in any country; and that HLD solutions have a substantial organizational and financial impact on care. 

## Figures and Tables

**Table 1:** Biological composition of samples used for artificial contamination.

Sample Infection	Human Papillomavirus (HPV) Genotype				
**Mono-Infected**	Sample 1: 16	Sample 2: 18	Sample 3: 31	Sample 4: 45	Sample 5: 51
**Bi-Infected**	Sample 6: 16–39	Sample 7: 16–56	Sample 8: 21–66	Sample 9: 52–66	
**Multi-Infected**	Sample 10: 16–51–59	Sample 11: 33–51–72	Sample 12: 16–40–66–83	Sample 13: 6–53–56–58–59	Sample 14: 16–18–39–51–53–66–82
**Non-Infected**	Sample 15: Negative				

**Table 2:** Comparison of chemical and ultraviolet disinfection. HPV-DNA found on one probe was positive with high-risk HPV 51 DNA. The difference was not statistically significant (NS) for HPV DNA but was significant for human DNA (*p* < 0.05).

Nature of the Remaining DNA	Chemical Disinfection	Ultra-Violet Disinfection	*p*-Value
**Human DNA**	7/47 (14.9%)	0/47 (0%)	0.0123
**HPV DNA**	1/47 (2.1%)	0/47 (0%)	NS

## Supplementary Materials

The following are available online at https://www.mdpi.com/2077-0383/8/11/1776/s1, Table S1: Biological screening for the second phase; Table S2: Biological screening for third phase.
