# Supplementary material for: Decontamination of Intravaginal Probes Infected by Human Papillomavirus (HPV) Using UV-C Decontamination System
Source: J Clin Med. 2019 Oct 24;8(11):1776. doi: 10.3390/jcm8111776 (PMC6912769; doi:10.3390/jcm8111776)
Supplement: Supplementary file 1 [file jcm-08-01776-s001.pdf]

Supplementary tables

Table S1. Biological screening for the second phase. * only viral results were presented for these first samples, as all samples were positive for human DNA. Viral genotypes were indicated in second and third column if appropriate.

Sample ID	Viral Results (probes cover) *	Results (After Wipe Cleaning)	Results (After AS1 Disinfection)
**21352258**	59/70	No viral DNA	No DNA
**21402081**	52/53/66	No viral DNA	No DNA
**21402084**	52/82	No viral DNA	No DNA
**21402085**	53	No viral DNA	No DNA
**21402235**	52	No viral DNA	No DNA
**21402236**	31/61	No viral DNA	No DNA
**21402238**	33	No viral DNA	No DNA
**21402239**	6	No viral DNA	No DNA
**21402240**	61	No viral DNA	No DNA
**21402242**	16	No viral DNA	No DNA
**21402417**	61	No viral DNA	No DNA
**21402423**	58	No viral DNA	No DNA
**21402497**	66	No viral DNA	No DNA
**21402515**	16	No viral DNA	No DNA
**21402605**	16/52/54/66	No viral DNA / Human DNA	No DNA
**21402689**	53	No viral DNA	No DNA
**21402690**	44	No viral DNA	No DNA
**21402691**	70	No viral DNA	No DNA
**21402794**	52	No viral DNA	No DNA
**21402806**	82	No viral DNA	No DNA
**40-197**	51	No viral DNA / Human DNA	No DNA
**40-060**	51	No viral DNA	No DNA
**39-148**	18	No viral DNA / Human DNA	No DNA
**40-061**	16	No viral DNA	No DNA
**21403340**	31/52	No viral DNA	No DNA
**21403342**	16	No viral DNA / Human DNA	No DNA
**21403343**	31	No viral DNA	No DNA
**21403344**	53/83	No viral DNA	No DNA
**21403345**	16	No viral DNA	No DNA
**21403346**	52/53	No viral DNA	No DNA
**21403347**	31/61	No viral DNA	No DNA
**21403348**	16/39/51/54	51	No DNA
**21403351**	58	No viral DNA	No DNA
**21403398**	18/52	No viral DNA	No DNA
**21403399**	6	No viral DNA	No DNA
**21403400**	54/61/66	No viral DNA	No DNA
**21403446**	82	No viral DNA	No DNA
**21403448**	53	No viral DNA	No DNA
**21403487**	52	No viral DNA	No DNA
**21403488**	81	No viral DNA	No DNA
**21403170**	35	No viral DNA / Human DNA	No DNA
**39-401**	16/61/84	No viral DNA	No DNA
**44-066**	45/53/59/84	No viral DNA	No DNA

**45-399**	31/42/54	No viral DNA / Human DNA	No DNA
**45-447**	16/18/33	No viral DNA	No DNA
**45-543**	42/51/59	No viral DNA	No DNA
**45-631**	6/35/51	No viral DNA / Human DNA	No DNA

Table S2. Biological screening for third phase.

Sample ID	Contaminated Medium	Results
**38-481**	6/53/56/58/59	No DNA
**39-149**	16/39	No DNA
**40-197**	16/18/39/51/53/66/82	No DNA
**38-478**	16/51/59	No DNA
**40-060**	52/66	No DNA
**39-148**	18	No DNA
**39-191**	45	No DNA
**40-061**	16	No DNA
**38-512**	16/56	No DNA
**39-189**	31	No DNA
**41-350**	51	No DNA
**41-573**	16/40/66/83	No DNA
**40-482**	21/66	No DNA
**41-645**	51/62/83	No DNA
**39-196**	33/51/72	No DNA
**Negative Control**	-	No DNA
